# Exogenous L-lactate administration in rat hippocampus increases expression of key regulators of mitochondrial biogenesis and antioxidant defense

**DOI:** 10.3389/fnmol.2023.1117146

**Published:** 2023-03-16

**Authors:** Mastura Akter, Haiying Ma, Mahadi Hasan, Anwarul Karim, Xiaowei Zhu, Liang Zhang, Ying Li

**Affiliations:** ^1^Department of Neuroscience, City University of Hong Kong, Kowloon, Hong Kong SAR, China; ^2^Department of Biomedical Sciences, City University of Hong Kong, Kowloon, Hong Kong SAR, China; ^3^School of Clinical Medicine, Li Ka Shing Faculty of Medicine, The University of Hong Kong, Kowloon, Hong Kong SAR, China; ^4^Department of Precision Diagnostic and Therapeutic Technology, City University of Hong Kong, Futian Research Institute, Shenzhen, Guangdong, China; ^5^Centre for Regenerative Medicine and Health, Hong Kong Institute of Science & Innovation, Chinese Academy of Sciences, Hong Kong, Hong Kong SAR, China; ^6^Centre for Biosystems, Neuroscience, and Nanotechnology, City University of Hong Kong, Kowloon, Hong Kong SAR, China

**Keywords:** hippocampus, lactate, mitochondrial biogenesis, oxidative stress, proteomics, SIRT3, PGC-1 alpha

## Abstract

L-lactate plays a critical role in learning and memory. Studies in rats showed that administration of exogenous L-lactate into the anterior cingulate cortex and hippocampus (HPC) improved decision-making and enhanced long-term memory formation, respectively. Although the molecular mechanisms by which L-lactate confers its beneficial effect are an active area of investigations, one recent study found that L-lactate supplementation results in a mild reactive oxygen species burst and induction of pro-survival pathways. To further investigate the molecular changes induced by L-lactate, we injected rats with either L-lactate or artificial CSF bilaterally into the dorsal HPC and collected the HPC after 60 minutes for mass spectrometry. We identified increased levels of several proteins that include SIRT3, KIF5B, OXR1, PYGM, and ATG7 in the HPC of the L-lactate treated rats. SIRT3 (Sirtuin 3) is a key regulator of mitochondrial functions and homeostasis and protects cells against oxidative stress. Further experiments identified increased expression of the key regulator of mitochondrial biogenesis (PGC-1α) and mitochondrial proteins (ATPB, Cyt-c) as well as increased mitochondrial DNA (mtDNA) copy number in the HPC of L-lactate treated rats. OXR1 (Oxidation resistance protein 1) is known to maintain mitochondrial stability. It mitigates the deleterious effects of oxidative damage in neurons by inducing a resistance response against oxidative stress. Together, our study suggests that L-lactate can induce expression of key regulators of mitochondrial biogenesis and antioxidant defense. These findings create new research avenues to explore their contribution to the L-lactate’s beneficial effect in cognitive functions as these cellular responses might enable neurons to generate more ATP to meet energy demand of neuronal activity and synaptic plasticity as well as attenuate the associated oxidative stress.

## 1. Introduction

L-Lactate is a metabolic end product of glycolysis and works as an energy substrate for various tissues including skeletal muscle, cardiac muscle, liver, and brain (Adeva-Andany et al., 2014). According to the astrocyte-neuron L-lactate shuttle (ANLS) hypothesis (Pellerin and Magistretti, 1994), L-lactate is produced by astrocytes through glycogenolysis and glycolysis and then transported into the neuronal cytoplasm through monocarboxylate transporter 2 (MCT2). There it can be converted to pyruvate by lactate dehydrogenase (LDH) during which NADH is also produced that can eventually result in increased expression of plasticity-associated genes by activating NMDA (Yang et al., 2014) and/or MAPK signaling pathway (Magistretti and Allaman, 2018; Margineanu et al., 2018). Pyruvate can enter the mitochondria and be processed through Kreb’s cycle and oxidative phosphorylation to generate ATP. The demand of ATP is high in active neurons to maintain various physiological activities including neural plasticity and memory formation (Magistretti and Allaman, 2018). Although the ANLS hypothesis suggests that the supply of L-lactate from the astrocytic glycogenolysis and/or glycolysis fuels this energy demand in neurons, other studies have provided conflicting experimental evidence. Díaz-García et al. demonstrated that neuronal stimulation in mouse hippocampal (HPC) slices or *in vivo* results in increased neuronal glycolysis and lactate production (Diaz-Garcia et al., 2017). Therefore, the authors argued that the energy demand during neuronal activation is fueled by glucose rather than imported L-lactate produced by the astrocytes. The authors also suggested that ANLS might function at rest but not during neuronal stimulation.

L-lactate is associated with several disease processes. In the context of tumor, it creates a conducive microenvironment for tumor cell growth and acts as an energy source for tumors including glioma (Reuss et al., 2021; Li et al., 2022). Lactate infusion was shown to induce panic attacks in humans although the mechanism remains poorly understood (Goetz et al., 1996). Lactic acidosis, which can result from increased level of lactate, was demonstrated to induce astrocyte swelling *in vitro* (Ringel et al., 2006). Increased brain L-lactate is associated with the development of brain edema in rats with chronic liver disease (Bosoi et al., 2014). Although considered a metabolic waste product with potentially negative effect for a long time since its discovery (Ferguson et al., 2018), emerging evidence now suggests that L-lactate protects the brain in several pathologic conditions, such as diabetic encephalopathy, Alzheimer’s disease, stroke, traumatic brain injury, and epilepsy (Cai et al., 2022). It has also been shown to play critical role in learning and memory (Newman et al., 2011; Harris et al., 2019; Vezzoli et al., 2020; Li et al., 2022). A previous study demonstrated that exogenous L-lactate administration into anterior cingulate cortex (ACC) or optogenetic activation of ACC astrocyte (that releases L-lactate) improves decision making in normal as well as visceral hypersensitive rats (Wang et al., 2017). In line with this, other studies showed that administration of L-lactate into HPC enhanced memory in rats whereas inhibition of astrocytic glycogenolysis or inhibition of astrocytic or neuronal MCTs in the HPC impairs memory formation (Newman et al., 2011; Suzuki et al., 2011). Although these studies demonstrated that L-lactate entry into the neuron is needed for its beneficial effect, the mechanism by which it confers its beneficial effect is not clearly understood. One possibility is that the L-lactate is used as an additional energy substrate for neurons to fuel the high energy demand during the cognitive process consistent with the ANLS hypothesis (Newman et al., 2011). However, based on the recent finding that ANLS is not the energy source during neuronal activation (Diaz-Garcia et al., 2017), it is possible that L-lactate might play other roles that could contribute to its beneficial effects on learning and memory irrespective of its use as an energy substrate. In fact, multiple evidence suggest that L-lactate can play role as signaling molecule in addition to being an energy substrate. A specific receptor for L-lactate called hydroxycarboxylic acid receptor 1 (HCAR1) was identified (Liu et al., 2009) which is a G_i_ protein coupled receptor (also called GPR81) that inhibit adenylyl cyclase and was shown to reduce excitability of cortical and hippocampal neurons upon activation (Herrera-Lopez and Galvan, 2018; de Castro Abrantes et al., 2019). Furthermore, presence of a L-lactate receptor that is positively linked to adenylyl cyclase has also been suggested based on the findings of L-lactate’s ability to mediate excitation of locus coeruleus neurons (which requires adenylyl cyclase activity) without entering into the neuron (Tang et al., 2014). However, the beneficial effect of L-lactate in cognitive function cannot be fully explained by transmembrane signaling through these receptors, as the entry of L-lactate into the neuron was shown to be required for the long-term memory formation (Suzuki et al., 2011). It suggests the existence of other possible roles of L-lactate in the cell that might confer its beneficial effect besides being used as an energy substrate. Taken together, these studies highlight the complexity in the L-lactate’s effect on cellular function.

As described before, despite L-lactate’s demonstrated beneficial role, the molecular mechanisms by which L-lactate exerts its beneficial effects are an active area of investigations. Advances in transcriptomics and proteomics provide opportunity to investigate changes in the expression of genes in an unbiased manner. One study investigated genome-wide transcriptional changes induced by L-lactate in cortical neuronal cell cultures and found increased expression of genes involved in MAPK signaling pathway and in synaptic plasticity (Margineanu et al., 2018). Another study in neuroblastoma cells (SH-SY5Y) and *C. elegans* discovered that L-lactate causes a mild Reactive Oxygen Species (ROS) burst that induces antioxidant defenses and pro-survival pathways, such as PI3K/AKT and endoplasmic reticulum chaperones (Tauffenberger et al., 2019). However, to the best of our knowledge, changes at proteomics level induced by the *in vivo* administration of L-lactate in the brain has not been investigated yet. Therefore, in order to further explore the molecular changes induced by L-lactate that might expand our understanding of the mechanism of its beneficial effect, we performed proteomic study of HPC of rats to identify differentially expressed proteins due to L-lactate treatment.

In proteomic analysis, we found that administration of exogenous L-lactate into HPC increases the expression of several proteins including SIRT3, KIF5B, OXR1, PYGM, ATG7, and CAMK2G. SIRT3 regulates mitochondrial functions and homeostasis and protects cells against oxidative stress (Shen et al., 2020; Zhang et al., 2020). SIRT3 has also been linked to memory (Kim et al., 2019; Liu et al., 2019, 2021) and several neurodegenerative diseases (Sidorova-Darmos et al., 2018). Further analysis identified increased expression of the key regulator of mitochondrial biogenesis (PGC-1α) and mitochondrial proteins (ATPB, Cyt-c) as well as increased mitochondrial DNA (mtDNA) copy number in the HPC of L-lactate treated rats. OXR1 is also known to maintain mitochondrial stability (Wu et al., 2016) and mitigate the deleterious effects of oxidative damage in neurons by inducing resistance response against oxidative stress (Volkert and Crowley, 2020). It is also a key player in neurodegenerative diseases (Volkert and Crowley, 2020). Increased PYGM could facilitate astrocytic glycogen mobilization into neuron to provide substrate for pentose phosphate pathway to increase antioxidant capacity. ATG7 is a mediator of autophagy that could facilitate removal of dysfunctional and damaged organelles. Together, our study suggests that L-lactate can induce expression of proteins related to mitochondrial biogenesis and antioxidant defense. These findings create new avenues for further investigations to explore their contribution to the L-lactate’s beneficial effect in cognitive functions.

## 2. Methods

### 2.1. Animal use and care

Total 28 male Sprague–Dawley rats weighting about 250–300 g were used in this study. All rats were housed in a standard laboratory facility (25°C, 50% humidity, 12-h light/dark cycle with light on at 7:00 AM). All animals were supplied by the Laboratory Animal Services Centre, Chinese University of Hong Kong. All experimental procedures using animals were conducted according to the guidelines developed by the Committee on Use and Care of Animals, Department of Health, Govt. Hong Kong SAR. The License numbers to conduct experiments are: (22–2) in DH/HT&A/8/2/5 Pt.8 and (22–3) in DH/HT&A/8/2/5 Pt.8. The approval for “Ethical Review of Research Experiments involving Animal Subjects” were taken by Animal Research Ethics Sub-Committee, City University of Hong Kong (References: A-0513 and A-0215).

### 2.2. Drug administration and isolation of HPC for proteomics

Rats were anesthetized with sodium pentobarbital (50 mg/kg, I.P.) and placed in a stereotaxic instrument (Kopf instrument). After exposing the skull, bilateral craniotomy (0.5–0.8 mm holes, 4 mm posterior to bregma and 2.6 mm lateral from midline) was done. In one group of rats (n = 5), each HPC (3.5 mm ventral from the surface of the skull at the craniotomy site) was infused with 1 μL of artificial cerebrospinal fluid (ACSF; NaCl 124 mM, KCl 3 mM, CaCl_2_ 2.4 mM, MgSO_4_ 1.3 mM, glucose 10 mM, and HEPES 10 mM, pH 7.3) at a flow rate of 0.1 μL/min (controlled by microinjection pump; World Precision Instruments, USA). In another group of rats (n = 5), 1 μL of 100 mM sodium L-lactate (Sigma Aldrich, Cat. #L7022) solution (sodium L-lactate dissolved in ACSF) was infused similarly per HPC. This is estimated to produce a final average concentration of 5 mM across the injection site and was shown to rescue 1,4-dideoxy-1,4-imino-D-arabinitol (DAB) induced impairment of long term memory formation in rat (Suzuki et al., 2011). Needle was kept at injection site for additional 5 minutes for the proper dispersion of solution after ACSF/L-lactate infusion. Then rats were kept in their cages for approximately 60 minutes. After that, a higher dose of sodium pentobarbital was given, and decapitation was done quickly. Following decapitation, the dura mater was removed carefully, and the brain was flipped out from skull into ice-cold PBS using spatula. The right and left HPC were isolated on an ice-cold plate using sterile forceps and were stored immediately at −80°C until further use. Later, the right HPC was used as sample for mass spectrometry (MS) whereas the left HPC was used for Western blot (WB) analysis.

### 2.3. Sample preparation for LC–MS/MS

#### 2.3.1. Homogenization

The right HPC was kept in 500 μL of lysis buffer containing 100 mM Tris–HCL (pH 7.4), 100 mM DTT, 4% SDS, protease inhibitors, and phosphatase inhibitors. Homogenization was done in the Precelly’s tissue homogenizer for three cycles with 30 s on and 30 s pauses. Then sonication was done on ice at 10% power for 16 cycles with 5 seconds on and 10 s off each cycle (Branson Digital Sonifier). The homogenates were then centrifuged at 21,000 × *g* at 4°C for 1 hour. The supernatant (150 μL) containing the total cell proteins was mixed with four times volume (600 μL) of ice-cold acetone for overnight precipitation at −20°C.

#### 2.3.2. Protein concentration determination

Protein precipitates were centrifuged at 14,000 × *g* for 30 min. The pelleted proteins were dissolved in 150 μL of 8 M urea and protein concentration was determined by Bradford assay. Volume that contains 20 μg protein was taken and Tris–HCL (pH 8.2) was added to it to make the total volume to 20 μL.

#### 2.3.3. Protein digestion

For the digestion of protein, the sample was first reduced by 20 mM DTT at 55°C for 1 hour and then alkylated by iodoacetamide in dark at room temperature for 40 minutes. Then the solution was diluted to 2 M urea by 50 mM Tris–HCl (pH 8.2). Then, trypsin was added at an enzyme/substrate ratio of 1:20 w/w and incubated at 37°C for overnight. On the second day, another same dose of trypsin was added to the reaction and incubated at 37°C for 1 hour. To stop the reaction, 10% formic acid (FA) was added to the reaction at a v/v ratio of 1:25. pH paper was used to make sure that pH is <2.0.

#### 2.3.4. Desalting

Sep-Pak C18 Vac cartridges, 50 mg (Waters, Milford, MA, USA) was washed with 50 μl of 80% ACN (acetonitrile) in 0.1% FA (centrifugation at 450 × *g* for 3 minutes). Then 50 μl of 0.1% FA in ddH_2_O was added to equilibrate the column (centrifugation at 450 × *g* for 3 minutes). After equilibration, samples were passed through the column twice. Then the column was washed again with 0.1% FA in ddH_2_O. Elution was done with 50 μL of 60% ACN in 0.1% FA. Then solution was dried by vacuum concentrator for 45 minutes. Dried peptides were then resuspended in 20 μl of 0.1% FA in ddH_2_O and centrifuged at 15,000 × *g* for 10 minutes. The solution was kept in −20°C until mass spectrometry.

### 2.4. LC–MS/MS analysis

LC–MS/MS analysis was done as described previously (Ma et al., 2021). The concentration was estimated by measuring absorbance at 280 nm (NanoDrop 2000, Thermo Fisher Scientific). All peptide samples were analyzed using a Q Exactive HF hybrid quadrupole-Orbitrap mass spectrometer coupled to an EASY-nLC™ 1,200 system (Thermo Fisher Scientific, Waltham, MA, United States). For each sample, 2 μL of peptide mixture was resolved using an analytical C18 column (250 mm, 75 μm, 3 μm; PepSep, Denmark) at a flow rate of 250 nl/min for 75 minutes. The mobile phase was a mixture of buffer A and buffer B (0.1% FA in 80% ACN) with a changing gradient of buffer B as follows: 0–2 minutes in 3%–7%; 2–52 minutes in 7%–25%; 52–62 minutes in 25%–44%; 62–70 minutes in 44%–95%; and 70–75 minutes in 95%. MS recording was operated in the range of 350–1,800 *m/z* with a mass resolution of 120,000. The positive ion mode was employed with the spray voltage at 2,500 V, and a spray temperature of 320°C. The resolution of dd-MS^2^ was 30,000 with a 1×10^5^ of AGC target. The Maximum IT was set at 60 ms and loop count was 12. The isolation window was 1.6 *m/z* and fixed first mass was 120.0 *m/z*.

### 2.5. Data processing and proteomic analysis

Data processing and analysis were done as described previously (Ma et al., 2021). Raw MS data were processed using the Proteome Discoverer software v2.2 and proteins were identified by searching MS/MS spectra against in tryptic digest of *Rattus norvegicus* (Rat) database downloaded from UniProt. The MS/MS spectra were searched with carbamidomethyl/ +57.021 Da (C) as static modification, as well as oxidation/ +15.995 Da (M), acetyl/ +42.011 Da (N-Teminus) as dynamic modifications. A maximum of two missed cleavages were allowed. The minimum peptide length was set to six amino acids and the maximum peptide length was set to 144 amino acids. The abundances of the protein were calculated by untargeted label-free quantification based on the precursor ion peaks across runs.

The quantified values of proteins were normalized based on the total peptide intensity of the samples to get the normalized abundances. Proteins with at least three valid values in all replicates were retained for bioinformatic analysis in R programming environment (R v4.0.3 in RStudio v1.3.1093). Missing values were imputed by median imputation. R package Limma (Ritchie et al., 2015) was used for protein differential expression analysis (*p* value <0.05 and log2 fold change >1 or < −1). The differentially expressed proteins were then visualized in a volcano plot using ggplot2 package (Wickham, 2009).

### 2.6. Western-blot analysis

The samples were homogenized in RIPA buffer containing (150 mM NaCl, 50 mM Tris pH 7.4, 1% Triton X-100, 0.1% SDS, 1% sodium deoxycholate), phosphatase and protease inhibitor cocktail (Sigma-Aldrich). Homogenates were then centrifuged at 14,000 × *g* for 30 min at 4°C. The supernatants were collected carefully as total protein and then Bradford assay (Bio-Rad cat. #5000205) was used to determine the protein concentration. Using sodium dodecyl sulfate-polyacrylamide gel electrophoresis (SDS-PAGE), 20 μg of proteins were separated, and then transferred to a polyvinyl fluoridene membrane (PVDF). Then the membrane was incubated with 5% non-fat milk in TBST (1X tris-buffered saline with 0.1% Tween® 20 Detergent) for 1 hour followed by incubation with primary antibodies with reference concentration for overnight at 4°C. Next day, membranes were washed for three times (5 minutes each) with TBST and incubated for 2 hours with horseradish peroxidase coupled secondary goat anti-rabbit or anti-mouse IgG (1:5000, Invitrogen) in TBST. Then membranes were washed three times (5 minutes each). Western Bright ECL HRP substrate (Advansta Inc., cat. #K12045-D20) was used to visualize the blot. Images were captured and processed by Chemidoc Touch Imaging System (Bio-Rad) and quantified by FIJI ImageJ software (National Institutes of Health, Bethesda, MD, USA). Expression of target proteins were normalized with that of β-actin or GAPDH level. Information of antibodies are given in Supplementary Table S1.

### 2.7. Immunohistochemistry and confocal microscopy

A separate (second) cohort of rats was used for IHC and confocal microscopy to validate proteomics results and to further investigate the hypotheses generated from HPC proteomics data. L-lactate (n = 5) or ACSF (n = 5) was administered as described before. After 60 minutes of injecting L-lactate/ACSF, rats were anesthetized by sodium pentobarbital (50 mg/kg, I.P.) and perfused transcardially with 0.9% saline for approximately 5 minutes and then perfused with 4% paraformaldehyde (PFA). The whole brain was taken out and postfixed in 4% PFA overnight at 4°C and cryoprotected in 30% sucrose dissolved in 1X PBS for an additional 3 days at 4°C. The brains were then stored in OCT medium at −80°C until further use. For IHC, each brain was sectioned at 40 μm using cryostat (Leica, USA) and processed as free-floating sections. Sections with dorsal HPC region were collected. Six to eight sections were selected from each rat for IHC. Sections were incubated with blocking solution of 10% normal goat serum (NGS) in PBS with 0.3% Triton X-100 for 1 hour at room temperature after a brief wash. Then sections were incubated with primary antibodies (listed in Supplementary Table S1) in blocking solution for overnight at 4°C. In the following day, slices were washed three times (5 minutes each) and incubated with targeted Alexa flour secondary antibodies (1: 300) in DAPI (1:20,000, Sigma) for 2 hours at room temperature. Then the sections were mounted into microscopic slides (Epredia™ SuperFrost Plus™ Adhesion Microscopic Slides) and covered with coverslips (Eprdia Cover Slip) along with fluorescent mounting medium (DAKO). The imaging was done by inverted laser scanning confocal microscope (LSM 880; Carl Zeiss, Oberkochen, Germany). The confocal images for quantitative analysis between two groups were acquired under 20×, 40× oil, and 63× oil objectives. The ratio between the intensity of fluorescence and area of analysis (mm^2^) was calculated using ImageJ and taken as quantitative expression of targeted immunofluorescence.

### 2.8. Relative mitochondrial DNA content quantification

Another cohort (third cohort) of rats was prepared for relative mtDNA content quantification. L-lactate (n = 4) or ACSF (n = 4) was administered as described before. After 60 minutes of injecting L-lactate/ACSF, rats were decapitated following higher dose of sodium pentobarbital. Total genomic DNA was extracted from hippocampus tissue using QIAamp DNA Mini Kits (cat. #51304) according to manufacturer’s protocol. Purity (A260/A280) and concentration of genomic DNA were checked with NanoDrop™ 1,000 Spectrophotometer (Thermo scientific). The DNA sample was stored at −20°C until further use.

Quantitative real-time PCR was performed with the SsoAdvanced Universal SYBR Green Supermix using Applied Biosystems QuantStudio™ 3 Real-Time PCR Systems. β-actin gene and mitochondrial D-loop were used as nuclear DNA (nDNA) and mtDNA, respectively, to investigate the abundance of mtDNA relative to nDNA. The primer sequences (Branda et al., 2002; Chou et al., 2007) and reaction mixture protocol are given in Table 1. Thermal cycling was done according to the SsoAdvanced Universal SYBR Green Supermix protocol. DNA from each rat was amplified as triplicate. After obtaining both mtDNA and nDNA Ct values from Real Time PCR software, we averaged the Ct values from triplicates for each rat. To determine the mtDNA content relative to the nDNA, the following equation (Rooney et al., 2015) was used:

**Table 1:** Preparation of 20 μL reaction mixture for real-time PCR.

Component with concentration	Volume added	Final concentration/ amount
2X, SsoAdvanced Universal SYBR Green Supermix (Bio-Rad)	10 μL	1X
5 ng/μL genomic DNA	2 μL	10 ng
Rat D-loop Forward Primer: 5′-GGTTCTTACTTCAGGGCCATCA-3′ (5 μM) [For mtDNA reaction]	2 μL	400 nM
Rat D-loop Reverse Primer: 5′-GATTAGACCCGTTACCATCGAGAT-3′ (5 μM) [For mtDNA reaction]	2 μL	400 nM
Rat β-actin Forward Primer: 5′-GGGATGTTTGCTCCAACCAA-3′ (5 μM) [For nDNA reaction]	2 μL	400 nM
Rat β-actin Reverse Primer: 5′-GCGCTTTTGACTCAAGGATTTAA-3′ (5 μM) [For nDNA reaction]	2 μL	400 nM
Nuclease free H_2_0	4 μL	

Relative mitochondrial DNA content = 2 × 2^(nDNA Ct – mtDNA Ct)^.

### 2.9. Statistical analysis

Statistical analyses were done with Prism v7.0 (GraphPad Software, La Jolla, CA, United States). Data are presented as mean ± SD as appropriate. Comparisons of IHC, WB, and relative mtDNA abundance between the experiment groups were done with two-tail unpaired Student’s *t*-test as appropriate with *p* value <0.05 for statistical significance.

## 3. Results

### 3.1. Identification of differentially expressed proteins in hippocampus following L-lactate infusion

**Figure 1:**
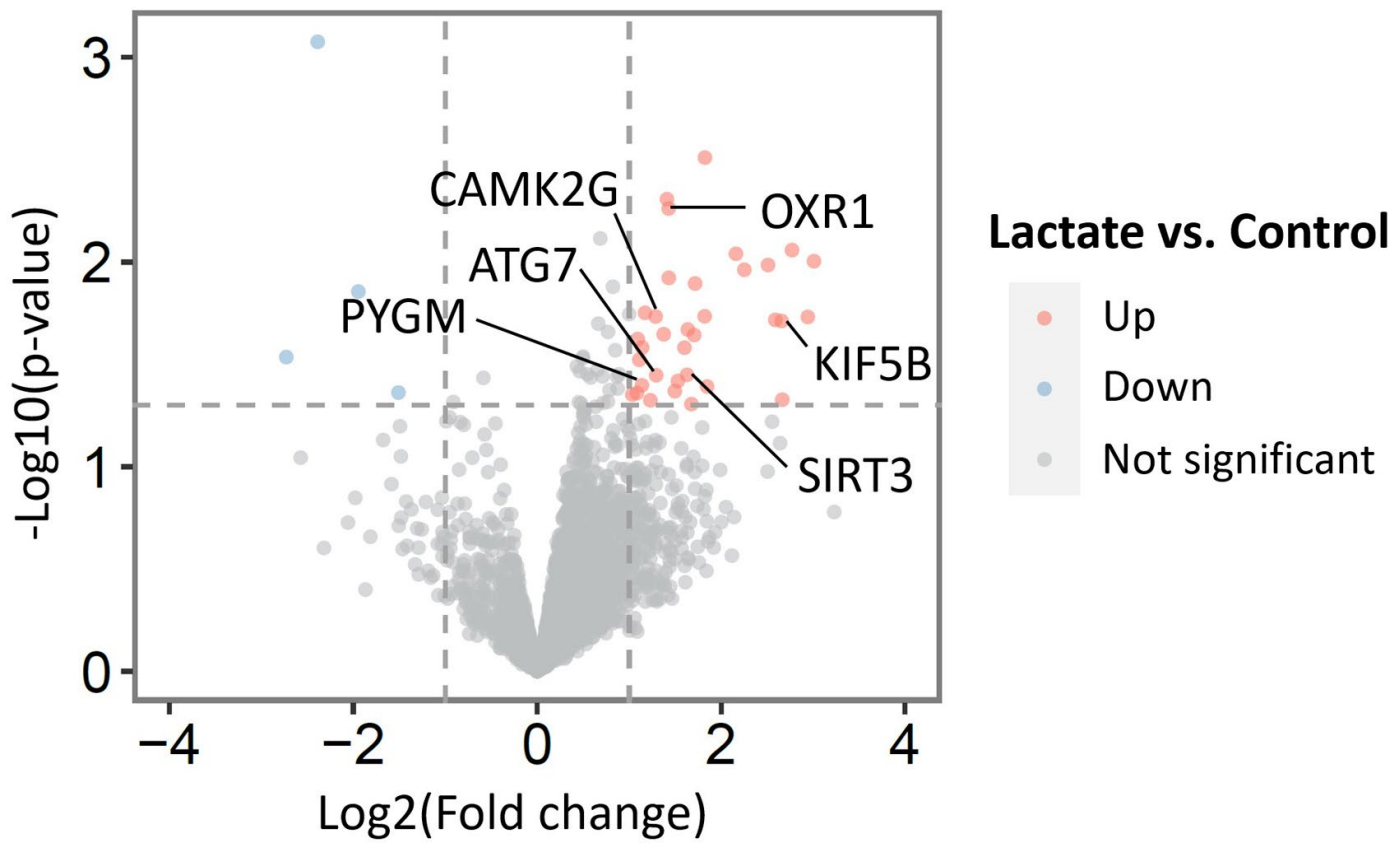
Volcano plot with all identified proteins from proteomic analysis of HPC of L-lactate treated rats and ACSF treated rats. Thirty four upregulated and four downregulated proteins were found in lactate group compared to control.

To investigate the effect of L-lactate in HPC at proteomics level, we prepared two groups of rats (first cohort). One group of rats (lactate group, *n* = 5) were infused with L-lactate (1 μL of 100 mM sodium L-lactate solution) into the dorsal HPC bilaterally as described in the method section. Similarly, another group of rats (control group, *n* = 5) were infused with ACSF bilaterally into the dorsal HPC. The rats were decapitated, and HPC of both sides were collected after 60 minutes of L-lactate/ACSF administration. The 60 minutes interval was chosen as we were primarily interested in investigating earlier molecular changes induced by L-lactate administration. Wang et al. administered L-lactate into ACC 15 minutes before starting the rat gambling test which is a 60-minutes test (Wang et al., 2017). They found that exogenous L-lactate improves decision-making. Therefore, we investigated if there are detectable molecular changes in proteomics within 60 min of L-lactate administration that might be associated with its beneficial effects in learning and memory. The HPC of right side was processed for LCMS and that of the left side was kept for western blot validation of selected proteins. During proteomics data analysis, one sample from the lactate group was excluded due to poor data quality. Therefore, we were left with four samples in lactate group and five samples in the control group for final data analysis. Then, only the proteins that were identified in at least three samples (i.e., three rats) in a group were considered for differential expression analysis. With this criterion, we identified a total of 2,617 proteins from all samples. From differential expression analysis, we identified 34 upregulated (including SIRT3, KIF5B, OXR1, PYGM, ATG7, and CAMK2G) and four downregulated proteins (Table 2; Figure 1) in lactate group compared to control. To validate the proteomics results, we performed WB analysis of selected proteins (SIRT3, RTN1, RPS6KA3) from the left sided HPC of same rats that were used for proteomics (Supplementary Figure S1). Consistent with the proteomics results, all the selected proteins showed increased expression in the L-lactate treated rats in WB analysis.

**Table 2:** Differentially expressed proteins in HPC in the L-lactate treated rats compared to controls.

Accession	Gene	Log2 FC	*p* value	Up/ Down	Protein
Q99MI7	*Uba3*	3.0091	0.0099	Up	NEDD8-activating enzyme E1 catalytic subunit
D3ZUU5	*Dnajb1*	2.9427	0.0186	Up	DnaJ (Hsp40) homolog, subfamily B, member 1 (Predicted), isoform CRA_a
F1M0X6	*Magohb*	2.7704	0.0088	Up	Mago homolog B, exon junction complex subunit
Q8R1R5	*Cd99l2*	2.6647	0.0470	Up	CD99 antigen-like protein 2
Q2PQA9	*Kif5b*	2.6576	0.0194	Up	Kinesin-1 heavy chain
Q5U2V8	*Emc3*	2.5886	0.0192	Up	ER membrane protein complex subunit 3
Q62991	*Scfd1*	2.5101	0.0103	Up	Sec1 family domain-containing protein 1
A0A0G2K226	*Nudcd3*	2.2524	0.0109	Up	NudC domain-containing 3
G3V963	*Tmem132a*	2.1615	0.0091	Up	RCG47487, isoform CRA_b
Q4VFZ4	*Katnb1*	1.8478	0.0406	Up	Katanin p80 WD40 repeat-containing subunit B1
Q9WVI4	*Gucy1a2*	1.8243	0.0031	Up	Guanylate cyclase soluble subunit alpha-2
M0R608	*Rtn1*	1.8214	0.0184	Up	Reticulon
A0A0G2JTK4	*Ppp6r1*	1.7173	0.0127	Up	Protein phosphatase 6, regulatory subunit 1
D3Z9D0	*RGD1306271*	1.7069	0.0227	Up	Similar to KIAA1549 protein
D3ZCZ9	*Ndufs6l1*	1.6771	0.0495	Up	NADH dehydrogenase [ubiquinone] iron–sulfur protein 6, mitochondrial
M0RB22	*Ptprd*	1.6380	0.0214	Up	Protein-tyrosine-phosphatase
C6ZII9	*Sirt3*	1.6290	0.0356	Up	NAD-dependent protein deacetylase
A0A0G2K7U5	*Adap1*	1.6010	0.0262	Up	ArfGAP with dual PH domains 1
F2Z3T7	*Isoc1*	1.5297	0.0381	Up	Isochorismatase domain-containing protein 1
F1LPG9	*Washc2c*	1.4967	0.0427	Up	WASH complex subunit 2C
A0A0G2K2W2	*Cmc4*	1.4311	0.0119	Up	Uncharacterized protein
A0A0G2K7Y2	*Oxr1*	1.4298	0.0055	Up	Oxidation resistance protein 1
Q05BA4	*Myadm*	1.4112	0.0049	Up	Myadm protein
A0A0G2KAT5	*Ptk2*	1.3751	0.0225	Up	Non-specific protein-tyrosine kinase
Q641Y5	*Atg7*	1.2948	0.0358	Up	Ubiquitin-like modifier-activating enzyme ATG7
D3Z8E0	*Rps6ka3*	1.2883	0.0184	Up	Ribosomal protein S6 kinase
F1LZX5	*Hectd4*	1.2304	0.0474	Up	HECT domain E3 ubiquitin protein ligase 4
F1M3F8	*Camk2g*	1.1729	0.0177	Up	Calcium/calmodulin-dependent protein kinase
Q32KK2	*Arsa*	1.1418	0.0262	Up	Arylsulfatase A
G3V8V3	*Pygm*	1.1386	0.0400	Up	Glycogen phosphorylase, muscle associated
F1M1B3	*Washc5*	1.1103	0.0300	Up	WASH complex subunit 5
A0A0G2JXP3	*Nedd4l*	1.0940	0.0238	Up	HECT-type E3 ubiquitin transferase
Q5U2U2	*Crkl*	1.0846	0.0437	Up	Crk-like protein
D3ZG88	*Znrd2*	1.0339	0.0445	Up	Mammary tumor virus receptor 2, isoform CRA_a
A0A0G2K7F7	*Tpm1*	−1.5082	0.0434	Down	Tropomyosin alpha-1 chain
F1M1A6	*LOC681355*	−1.9443	0.0139	Down	Similar to potassium channel tetramerisation domain-containing 12b
Q6QI86	*Hdhd2*	−2.3861	0.0008	Down	Immediate early response 3-interacting protein 1
P69736	*Edf1*	−2.7270	0.0291	Down	Endothelial differentiation-related factor 1

We performed PANTHER overrepresentation test for Reactome pathways using the gene list of the upregulated proteins (Ashburner et al., 2000; Mi et al., 2019; Gene Ontology, 2021; Thomas et al., 2022). The pathways that had raw *p* value <0.05 is provided in the Supplementary Table S2. These pathways include “FOXO-mediated transcription,” “mitochondrial biogenesis,” “glycogen metabolism,” “activation of NMDA receptors and postsynaptic events,” and “MAPK family signaling cascades.” We also performed protein–protein interaction (PPI) analysis with STRING (Szklarczyk et al., 2019) with whole genome background as shown in Supplementary Figure S2. Although the PPI enrichment *p* value for the whole set was not statistically significant (*p* value = 0.249), two potentially important interaction clusters were observed: one with SIRT3, OXR1, ATG7, and UBA3; another with CAMK2G, PTK2, and CRK2.

### 3.2. L-lactate infusion increases expression of SIRT3 in neurons of HPC

As mentioned before, among the upregulated proteins in L-lactate treated rats was SIRT3 (Sirtuin 3). SIRT3, a member of sirtuin family, is an NAD^+^-dependent protein deacetylase. Full-length SIRT3 (~43 kDa) which is the minor form and thought to be enzymatically inactive is proteolytically cleaved near the N-terminus in the mitochondrial matrix to become the short form (~28 kDa) which is the main and active from of SIRT3 (Zhang et al., 2020). While some studies suggested that SIRT3 protein is exclusively mitochondrial irrespectively of the expression level (Cooper and Spelbrink, 2008; Hallows et al., 2008), others detected variable expression of the long form of SIRT3 in addition to the short form (Baeken et al., 2021). In our WB experiment (Supplementary Figures S1A,B), we detected increased expression of the short form of SIRT3 but could not detect any band around 43 kDa (the immunogen for the antibody we used is a synthetic peptide corresponding to a sequence within amino acids 300 to the C-terminus of human SIRT3).

Previous study showed that exercise increases SIRT3 expression in hippocampal neurons (Cheng et al., 2016). Another study demonstrated that exercise or exogenous L-lactate promotes SIRT1 (another member of sirtuin family) expression in HPC and is involved in learning and memory (El Hayek et al., 2019). *Sirt3* knockout mice was shown to exhibit poor remote memory (Kim et al., 2019). On the other hand, overexpression of SIRT3 in the CA1 region of HPC in mice was shown to rescue anesthesia/surgery-induced learning and memory dysfunction (Liu et al., 2021). Taken together, these studies and our current finding of increased SIRT3 expression in HPC due to L-lactate infusion makes SIRT3 an interesting candidate for further investigation.

**Figure 2:**
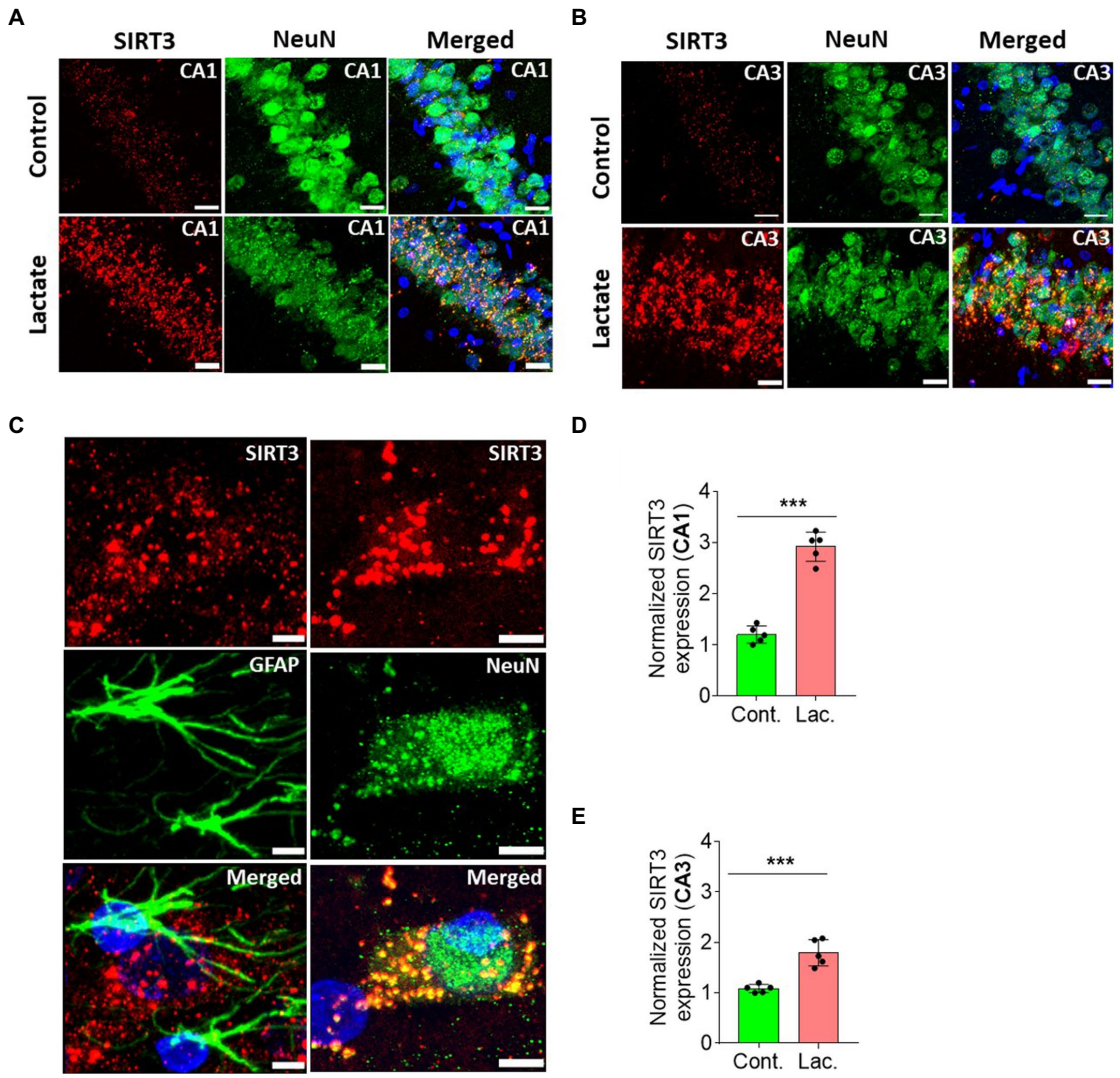
L-lactate causes increased SIRT3 expression in neurons of HPC. **(A,B)** Representative confocal micrograph of SIRT3 (red) co-labelled with NeuN (green) in the CA1 **(A)** and CA3 **(B)** areas of HPC of control and lactate groups. L-lactate infusion increased SIRT3 expression in both areas. Scale bars: 20 μm. **(C)** Representative confocal micrograph of SIRT3 (red) co-labelled with GFAP (green) or NeuN (green) in the HPC of lactate group. Scale bars: 5 μm. **(D,E)** Fluorescence intensity of SIRT3 stained sections in the CA1 (D) and CA3 **(E)** areas of HPC of lactate group was assessed and normalized to control group of rats. Data is shown as mean ± SD (*n* = 5 rats per group). p*** < 0.001, unpaired Student’s *t*-test.

A second cohort of rats was prepared (five rats in control group, and five rats in lactate group) similar to proteomics experiment to do IHC analysis of SIRT3 and other relevant proteins (described later). Our IHC analysis from this cohort of rats confirmed increased SIRT3 expression (in CA1 and CA3 neurons, but not in dentate gyrus) in L-lactate treated rats compared to ACSF (Figure 2; Supplementary Figure S3).

### 3.3. L-lactate infusion is associated with increased pCREB and PGC-1α expression in neurons of HPC

**Figure 3:**
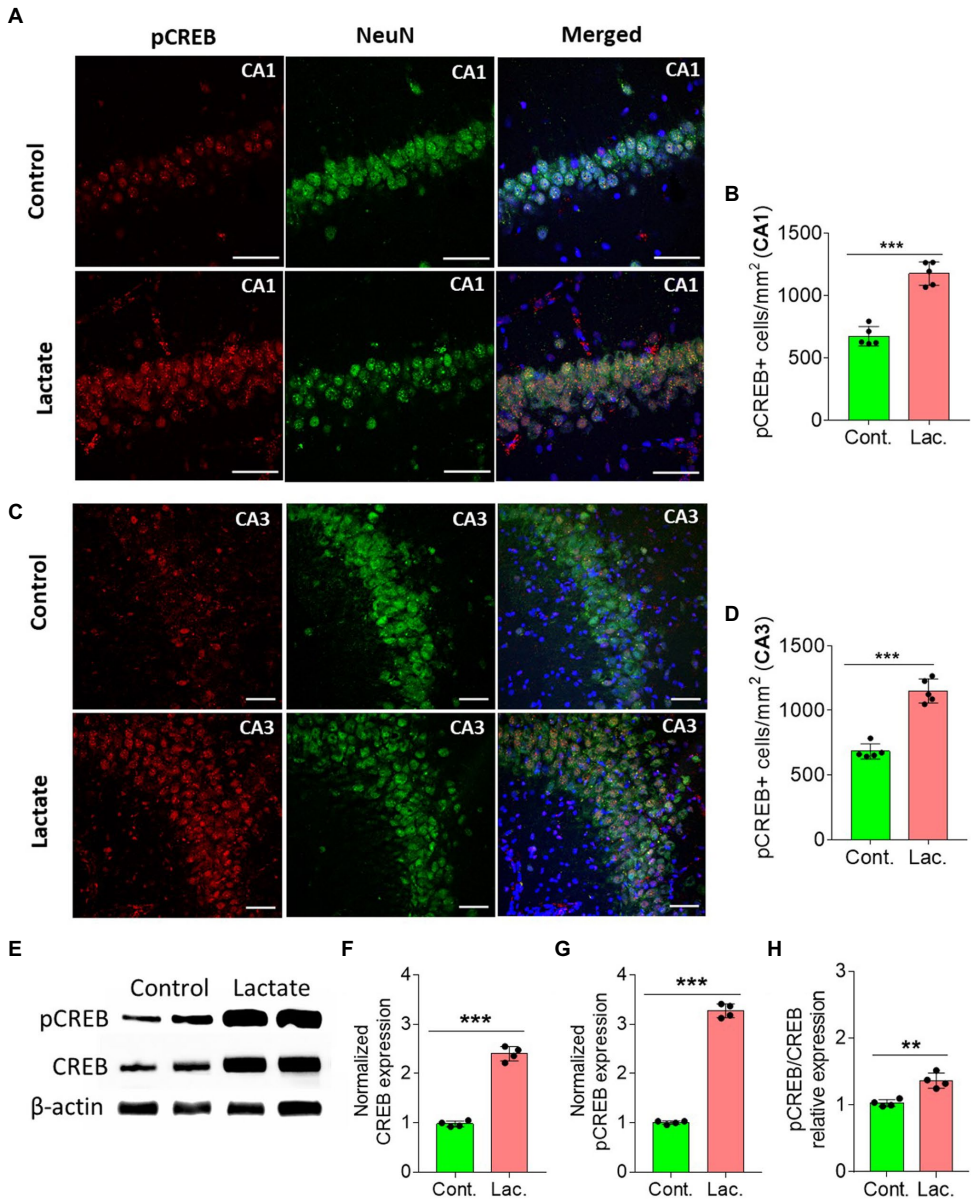
L-lactate increases CREB, pCREB, and pCREB/CREB in HPC. **(A,C)** Representative confocal micrograph of pCREB (red) co-labelled with NeuN (green) in the CA1 **(A)** and CA3 **(C)** areas of HPC of control and lactate groups. L-lactate infusion increased pCREB in both areas of HPC. Scale bars: 20 μm. **(B,D)** Number of pCREB+ cells in CA1 **(B)** and CA3 **(D)** areas of HPC of control and lactate groups. Data are shown as mean ± SD (*n* = 5 rats per group). p*** < 0.001, unpaired Student’s *t*-test. **(E–H)**. Representative WB images of pCREB and CREB in the HPC extracts from control and lactate groups. Intensity of pCREB and CREB was quantified and normalized with β-actin and was found to be significantly increased in lactate group compared to control **(F,G)**. pCREB/CREB ratio was also increased **(H)**. Data are shown as mean ± SD (*n* = 4 rats per group). p*** < 0.001, p** < 0.01, unpaired Student’s *t*-test.

Astrocytic glycogen breakdown and astrocyte-neuron L-lactate transport in HPC of rats was shown to be critical for long term memory formation and induction of memory related proteins including pCREB (a transcription factor) (Suzuki et al., 2011). pCREB is known to be involved in neurogenesis resulting in improvement of learning and memory (Ortega-Martinez, 2015). On the other hand, SIRT3 was shown to stimulate the phosphorylation of CREB in mice skeletal muscles (Shi et al., 2005; Palacios et al., 2009), whereas *Sirt3* knockout mice have decreased phosphorylation of CREB in skeletal muscles (Palacios et al., 2009). SIRT3 deacetylates and activates mitochondrial FOXO3A which then translocates to nucleus to indirectly phosphorylate the CREB (Sidorova-Darmos et al., 2018). Therefore, we hypothesized that L-lactate induced increase in SIRT3 in HPC in our study might also be associated with increased pCREB. We performed IHC analysis and found that pCREB immunoreactivity was significantly increased in neurons of CA1 and CA3 areas of HPC in L-lactate treated rats compared to control rats (Figures 3A–D). WB analysis showed increased level of pCREB, CREB and pCREB/CREB (Figures 3E–H).

**Figure 4:**
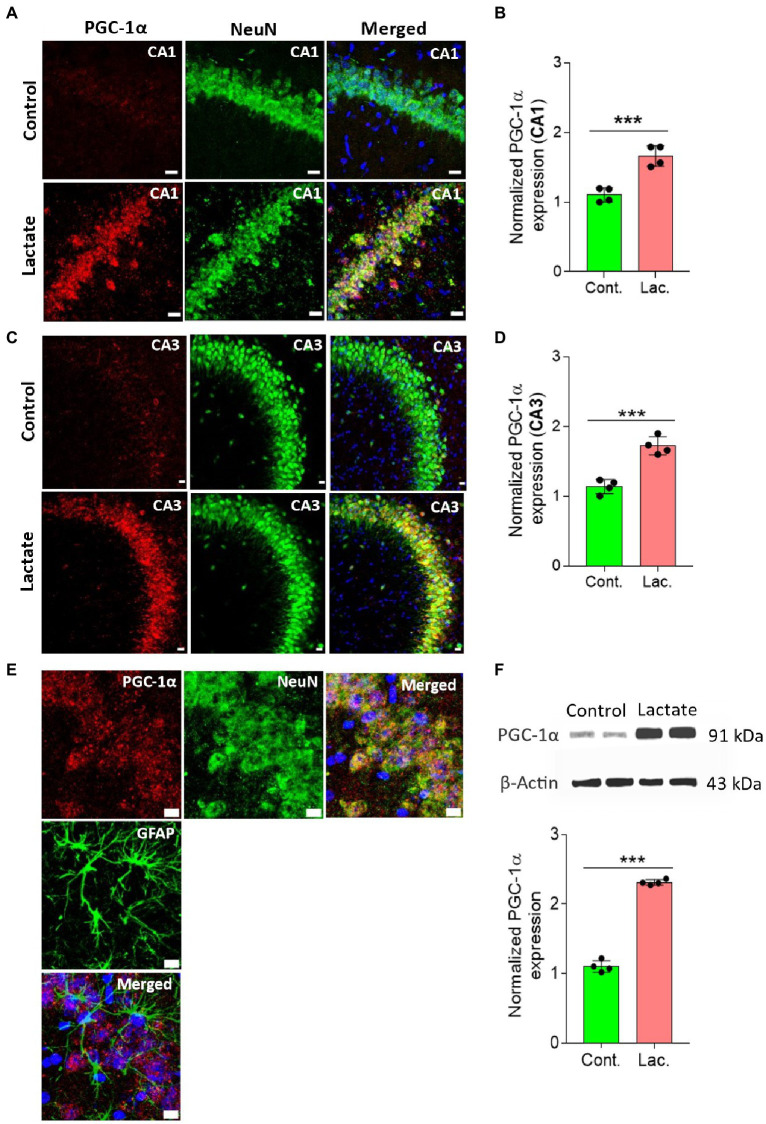
L-lactate increases PGC-1α expression in neurons of HPC. **(A,C)** Representative confocal micrograph of PGC-1α (red) co-labelled with NeuN (green) in the CA1 **(A)** and CA3 **(C)** areas of HPC of control and lactate groups. L-lactate infusion increased PGC-1α expression in both areas of HPC. Scale bars: 20 μm. **(B,D)** Fluorescence intensity of PGC-1α stained sections in the CA1 **(B)** and CA3 **(D)** areas of HPC of lactate group was assessed and normalized to control group. Data are shown as mean ± SD (n = 4 rats per group). p*** < 0.001, unpaired Student’s *t*-test. **(E)** Representative confocal micrograph of PGC-1α (red) co-labelled with GFAP (green) or NeuN (green) in the HPC of lactate group. Scale bars: 10 μm. **(F)** Representative WB images of PGC-1α in the HPC extracts from control and lactate groups. Intensity of PGC-1α was quantified and normalized with β-actin. PGC-1α was found to be significantly increased in lactate group compared to control. Data are shown as mean ± SD (*n* = 4 rats per group). p*** < 0.001, unpaired Student’s *t*-test.

CREB is known to increase PGC-1α expression by increasing its promoter activity (Herzig et al., 2001; Handschin et al., 2003). PGC-1α is a key regulator of mitochondrial biogenesis (Liang and Ward, 2006; Austin and St-Pierre, 2012). Therefore, we investigated whether PGC-1α is also increased in the L-lactate treated rats. Our IHC analysis shows that PGC-1α expression was significantly increased in neurons of CA1 and CA3 areas of HPC in L-lactate treated rats compared to control rats (Figures 4A–E). Furthermore, WB analysis of HPC from the first cohort of rats also confirmed increased expression of PGC-1α in L-lactate treated rats (Figure 4F), although the protein was not detected from proteomics data analysis.

### 3.4. L-lactate infusion induces mitochondrial biogenesis in neuron

L-lactate plays important roles in mitochondrial biogenesis (Kitaoka et al., 2016; Takahashi et al., 2019). Overexpression of neuronal L-lactate transporter MCT2 in HPC was found to be associated with increased mitochondrial biogenesis and recovery of cognitive impairment a rat model of stroke (Yu et al., 2021). Exogenous L-lactate administration or high-intensity exercise-induced L-lactate release from skeletal muscle was shown to promote hippocampal PGC-1α expression and mitochondrial biogenesis in mice (Park et al., 2021). On the other hand, overexpression of SIRT3 enhanced mitochondrial function and metabolism (Liu et al., 2018), whereas SIRT3 silencing causes decreased mitochondrial biogenesis (Torrens-Mas et al., 2019). SIRT3 is known to increase the efficiency of electron transport chain (ETC) of mitochondria by deacetylating and regulating activities of mitochondrial complexes I-V to increase ATP production (Sidorova-Darmos et al., 2018). Interestingly, in proteomic analysis we found increased expression of NDUFS6L1 (NADH dehydrogenase (ubiquinone) Fe-S protein 6, mitochondrial) in the L-lactate treated rats. NDUFS6L1 is a subunit of complex I of ETC. Taken together, our findings of increased SIRT3, PGC-1α, and NDUFS6L1 point toward increased mitochondrial biogenesis due to the L-lactate administration.

**Figure 5:**
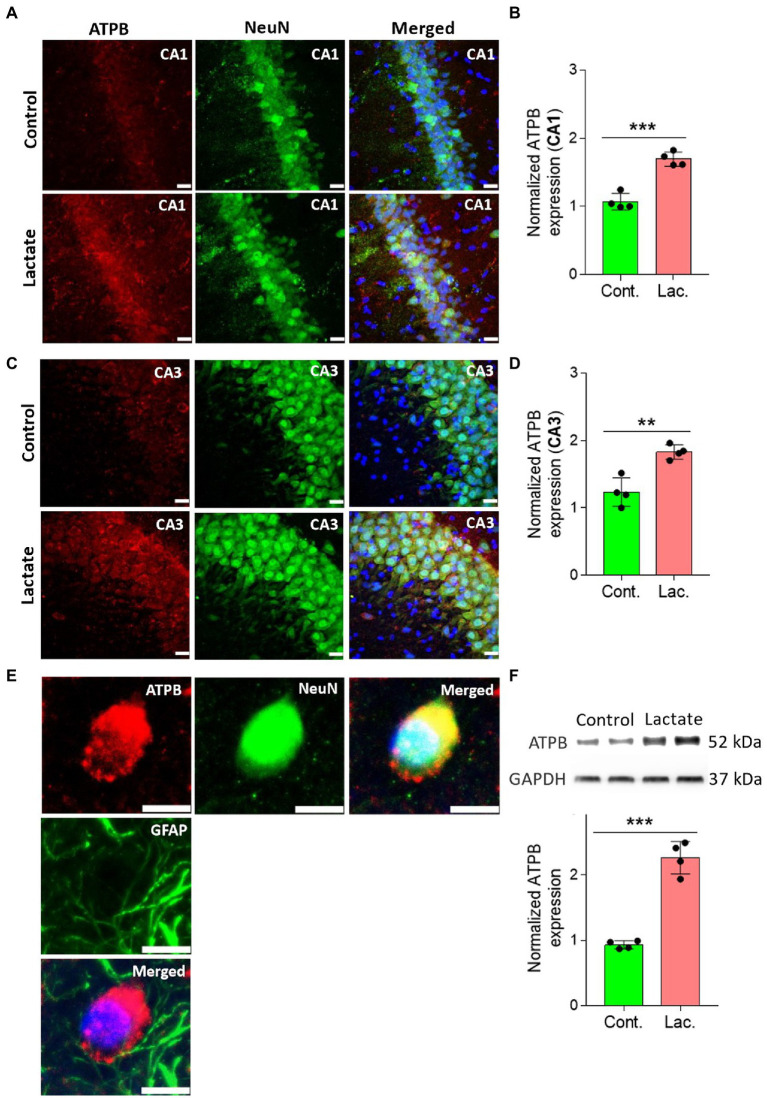
L-lactate increases ATPB expression in neurons of HPC. **(A,C)** Representative confocal micrograph of ATPB (red) co-labelled with NeuN (green) in the CA1 **(A)** and CA3 **(C)** areas of HPC of control and lactate groups. L-lactate infusion increased ATPB expression in both areas of HPC. Scale bars: 20 μm. **(B,D)** Fluorescence intensity of ATPB stained sections in the CA1 **(B)** and CA3 **(D)** areas of HPC of lactate group was assessed and normalized to control group. Data are shown as mean ± SD (*n* = 4 rats per group). p*** < 0.001, unpaired Student’s *t*-test. **(E)** Representative confocal micrograph of ATPB (red) co-labelled with GFAP (green) or NeuN (green) in the HPC of lactate group. Scale bars: 10 μm. **(F)** Representative WB images of ATPB in the HPC extracts from control and lactate groups. Intensity of ATPB was quantified and normalized with GAPDH. ATPB was found to be significantly increased lactate group compared to control. Data are shown as mean ± SD (*n* = 4 rats per group). p*** < 0.001, unpaired Student’s *t*-test.

**Figure 6:**
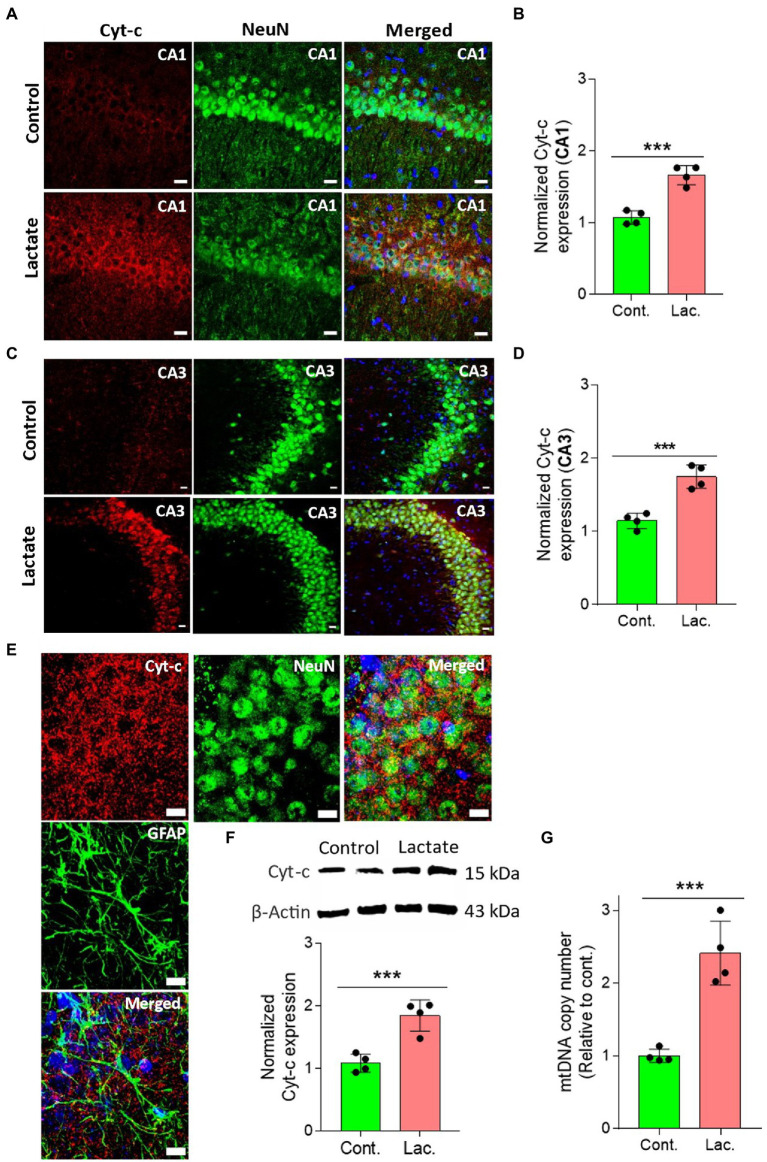
L-lactate increases Cyt-c expression in neuron of HPC and increases mtDNA copy number. **(A,C)** Representative confocal micrograph of Cyt-c (red) co-labelled with NeuN (green) in the CA1 **(A)** and CA3 **(C)** areas of HPC of control and lactate groups. L-lactate infusion increased Cyt-c expression in both areas of HPC. Scale bars: 20 μm. **(B,D)** Fluorescence intensity of Cyt-c-stained sections in the CA1 **(B)** and CA3 **(D)** areas of HPC of lactate group was assessed and normalized to control group. Data are shown as mean ± SD (*n* = 4 rats per group). p*** < 0.001, unpaired Student’s *t*-test. **(E)** Representative confocal micrograph of Cyt-c (red) co-labelled with GFAP (green) or NeuN (green) in the HPC of lactate group. Scale bars: 10 μm. **(F)** Representative WB images of Cyt-c in the HPC extracts from control and lactate groups. Intensity of Cyt-c was quantified and normalized with β-actin. Cyt-c was found to be significantly increased in lactate group compared to control. Data are shown as mean ± SD (*n* = 4 rats per group). p*** < 0.001, unpaired Student’s *t*-test. **(G)** mtDNA copy number abundance in the HPC of control and lactate groups relative to nDNA. Relative mtDNA copy number was significantly increased in lactate group compared to control. Data are shown as mean ± SD (*n* = 4 rats per group). p*** < 0.001, unpaired Student’s *t*-test.

To further confirm this, we performed IHC and WB analysis of ATPB (Mitochondrial ATP synthase subunit beta) and Cyt-c (Cytochrome c) which are components of oxidative phosphorylation system of mitochondria. ATPB is a part of mitochondrial membrane ATP synthase (Complex V) that produces ATP from ADP (Jonckheere et al., 2012). Cyt-c is located at the inner mitochondrial membrane where it functions as an electron shuttle between complex III and complex IV (Garrido et al., 2006). As shown in Figures 5, 6, ATPB and Cyt-c expression were significantly increased in neurons of CA1 and CA3 areas of HPC in L-lactate treated rats compared to control rats.

Finally, we performed mtDNA copy number analysis relative to nDNA and found increased mtDNA copy number in the HPC of L-lactate treated rats (Figure 6G). Increased relative mtDNA copy number abundance together with the increased expression of PGC-1α (a master regulator of mitochondrial biogenesis) are suggestive of increased mitochondrial biogenesis (Kong et al., 2010; Tseng et al., 2013; Torrens-Mas et al., 2017; Park et al., 2021; Yu et al., 2021). Therefore, taken together the observation of the increased expression of PGC-1α, increased mtDNA, and the increased mitochondrial proteins (ATPB, Cyt-c), our study suggests that exogenous L-lactate infusion into the HPC induces mitochondrial biogenesis in neuron.

## 4. Discussion

Brain is a highly energy demanding organ that uses 20% of total body oxygen consumption and 25% of total body glucose utilization despite being only 2% of the total body weight. Neurons use approximately 75%–80% of the energy produced in the brain (Berndt and Holzhutter, 2013; Watts et al., 2018). Neuronal action potential increases its ATP demand by tenfold (Berndt and Holzhutter, 2013). Mitochondria is the primary site of energy generation in the form of ATP by TCA cycle and oxidative phosphorylation. Oxidative phosphorylation is driven by the electrochemical gradient generated across the inner mitochondrial membrane due to the transfer of electron along the electron transport chain. However, this process also generates ROS due to “leak” of some electrons especially at complex I and III of the ETC (Beckhauser et al., 2016). In fact, mitochondria is the site of an estimated 90% of cellular ROS production (Balaban et al., 2005). ROS production is necessary for synaptic plasticity and long-term potentiation which plays important role in learning and memory (Massaad and Klann, 2011; Beckhauser et al., 2016). However, excess ROS have detrimental effect in these processes and is linked to memory impairment (Fukui et al., 2001; Hritcu et al., 2009), age-related decline in cognitive function, and pathogenesis of neurodegenerative diseases including Alzheimer’s disease and Parkinson’s disease (Massaad and Klann, 2011; Beckhauser et al., 2016). Therefore, ROS acts as double-edged sword in respect to memory formation. There exists multiple defense mechanisms in the cells to keep the ROS in check (Massaad and Klann, 2011). Excess ROS leads to oxidative stress which can induce damage to the mtDNA, mitochondrial membrane and respiratory chain, and alter Ca^2+^ homeostasis contributing to neurodegeneration (Guo et al., 2013). On the other hand, mitochondrial biogenesis reduces oxidative damage and increases ATP production (Ho et al., 2022). Previous study showed that exercise-induced release of L-lactate increases hippocampal PGC-1α mRNA expression and mtDNA copy number in mice (Park et al., 2021). Furthermore, L-lactate was shown to induce PGC-1α expression and mitochondrial biogenesis in L6 cell (Hashimoto et al., 2007). Interestingly, L-lactate was shown to produce a mild ROS burst that could induce mitochondrial biogenesis (Hashimoto et al., 2007) and promote oxidative stress resistance (Tauffenberger et al., 2019). In line with these findings, we also found increased expression of the key regulator of the mitochondrial biogenesis PGC-1α, components of the mitochondrial respiratory chain (ATPB and Cyt-c in WB, NDUFS6L1 in proteomics), and increased mtDNA copy number in the HPC of L-lactate treated rats compared to the controls.

In this study, we have demonstrated increased expression of SIRT3 in the HPC of L-lactate treated rats. SIRT3 is an NAD^+^-dependent protein deacetylase that is found in mitochondria and controls several metabolic processes including TCA cycle, respiratory chain, β-oxidation of fatty acids, and ketogenesis (Giralt and Villarroya, 2012). Its expression can be triggered by increased ROS. Induction of mitochondrial oxidative stress was shown to induce SIRT3 mRNA expression in primary hippocampal culture and its overexpression was found to be neuroprotective (Weir et al., 2012). SIRT3 upregulates the antioxidant defense system, promotes mitochondrial biogenesis by promoting PGC-1α expression, stabilizes mitochondrial membrane, and maintains function of the ETC. It reduces ROS generation and protects against oxidative, excitotoxic, and metabolic stress (Cheng et al., 2016; Shen et al., 2020; Sun et al., 2021). Interestingly, several recent studies have demonstrated that SIRT3 plays important role in learning and memory (Kim et al., 2019; Liu et al., 2019, 2021). These findings raise the possibility that SIRT3 might be contributing to the L-lactate’s beneficial effects in learning and memory observed in the previous studies (Newman et al., 2011; Harris et al., 2019; Vezzoli et al., 2020) by promoting mitochondrial biogenesis and antioxidant defense. These could enable the neurons to generate more ATP while reducing oxidative stress during bioenergetic challenges. Another interesting finding from the proteomics study was the increased expression of KIF5B in the hippocampus of L-lactate-treated rats. KIF5B is the main kinesin motor driving anterograde mitochondrial axonal transport in neuron (Lackner, 2014; Cromberg et al., 2019). Previous studies showed that KIF5B is crucial for dendritic spine morphogenesis, synaptic plasticity, and memory formation (Cai et al., 2007; Zhao et al., 2020). Therefore, it appears that L-lactate not only induces mitochondrial biogenesis but also regulates its’ microtubule-based transport system necessary for synaptic plasticity.

We also found increased expression of OXR1, PYGM, and ATG7 in the HPC of L-lactate treated rats. OXR1 (Oxidation resistance protein 1) is induced by oxidative stress and is localized to the mitochondria and cytoplasm (Volkert and Crowley, 2020). It controls the expression of oxidative stress resistance genes by acting on their regulatory elements and is known to mitigate neurodegeneration by reducing the damage and cell death caused by oxidative stress (Volkert and Crowley, 2020). PYGM (Glycogen phosphorylase, muscle associated) is one of the three isoforms of the glycogen phosphorylase. Other two isoforms are: PYGB (brain) and PYGL (liver). Glycogen phosphorylase is the rate-limiting enzyme of glycogenolysis that generates glucose-1-phosphate (G1P) from glycogen. In the brain, astrocytes express both PYGB and PYGM whereas neurons only express PYGB (Mathieu et al., 2019). Therefore, the increased expression of PYGM due to L-lactate administration suggests increased astrocytic glycogenolysis. Previous study in astroglia-rich primary cultures derived from rat brain showed that H_2_O_2_-induced stress stimulates mobilization of glycosyl residues from astrocytic glycogen to be used in the pentose phosphate pathway (PPP) to regenerate NADPH (Rahman et al., 2000). NADPH is required to regenerate the antioxidant glutathione (GSH) to eliminate ROS and their toxic byproducts (Rahman et al., 2000; Currais and Maher, 2013). Therefore, a plausible explanation for increased expression of PYGM in our study is to increase the antioxidant defense capacity in the neuron by mobilizing glucose residues from astrocytic glycogen into the neurons to feed PPP to enhance regeneration of GSH. ATG7 (Ubiquitin-like modifier-activating enzyme ATG7) is a core ATG protein that drives crucial stages of classical degradative autophagy through ATG8 lipidation (Collier et al., 2021). Autophagy plays crucial role in the removal of dysfunctional organelles which is especially important in maintaining normal functions of cells including neurons (Collier et al., 2021; Sukhorukov et al., 2021). Mitochondrial proteins in *Atg7* null flies demonstrated significantly prolonged half-lives compared with controls suggesting that *Atg7* also promotes mitophagy as a part of general autophagy (Vincow et al., 2013). ATG7 was found to contribute to mitochondrial clearance in erythroblasts and reticulocytes in mice (Zhang et al., 2009; Mortensen and Simon, 2010). Similarly, ATG7-deficient vascular smooth muscle cells showed impaired mitophagy (Nahapetyan et al., 2019). Tan et al. recently demonstrated a degradative to secretory autophagy switch mediating mitochondrial clearance in the absence of the ATG8-conjugation machinery in *ATG7* knockout HeLa cells and mice muscle (Tan et al., 2022). On the other hand, another recent study in mice showed that elevated lactate level due to high-intensity interval training does not affect mitophagy in hippocampus (Zhang et al., 2021). Therefore, although exogenous L-lactate induced increased ATG7 observed in our study might suggest induction of degradative autophagy, it remains unclear whether mitophagy is induced due to exogenous L-lactate treatment as ATG7 is itself not a specific marker of mitophagy. Further studies are needed to clearly elucidate this.

Our proteomic analysis of hippocampus also detected increased expression of proteins related to activation of NMDA receptors (CAMK2G) and MAPK family signaling cascades (CAMK2G, DNAJB1, PTK2) due to L-lactate administration into HPC. Previous studies showed that NADH, which can be produced from NAD^+^ during the conversion of L-lactate to pyruvate after entry into the neurons, can enhance the expression of plasticity-associated genes by activating NMDA (Yang et al., 2014) and/or MAPK signaling pathway (Magistretti and Allaman, 2018; Margineanu et al., 2018). NMDA receptor activation results in enhanced Ca^2+^ currents that could lead to enhanced CaMKII (Calcium/calmodulin-dependent protein kinase) signaling which plays crucial role in synaptic plasticity, learning and memory (Yasuda et al., 2022). Ca^2+^ currents and CaMK signals are also important stimuli for mitochondrial biogenesis through pCREB/PGC-1α (Cardanho-Ramos and Morais, 2021).

In summary, our study suggests that L-lactate increases the expression of key regulators of mitochondrial biogenesis and antioxidant defense. Future studies could be aimed at delineating how and to what extent these might contribute to the L-lactate’s beneficial effects in cognitive functions.

### 4.1. Future directions

The current study has identified several differentially expressed proteins in the HPC of rats due to exogenous L-lactate administration. Although we have discussed that the expression of several of these proteins might be related to the L-lactate induced mild ROS burst (Tauffenberger et al., 2019), further studies are needed to delineate the molecular mechanisms (e.g., recent study (Zhang et al., 2019) identified histone lactylation as a novel mechanism of L-lactate-regulated gene expression) of their differential expressions and their downstream beneficial or detrimental effects on brain.

Study showed that mitochondria from *Sirt3*−/− mice display reduced complex I activity whereas incubation of exogenous SIRT3 with mitochondria can augment Complex I activity. The decreased activity and basal ATP production in *Sirt3*−/− mice was mediated by the increased acetylation of the components of the complex I (Ahn et al., 2008). Therefore, it is reasonable to hypothesize that in the HPC of the L-lactate treated rats, the activity of the complex I might be increased due to increased SIRT3 that might deacetylate the components of complex I. It would be interesting to investigate the changes in the expression levels as well as the acetylation status of the components of the complex I due to L-lactate treatment. Several mitochondrial acetylome studies have characterized and cataloged the SIRT3 substrates from liver tissue (Fritz et al., 2012; Hebert et al., 2013; Rardin et al., 2013). However, similar dataset in the context of neuron or brain tissue is scarce. Therefore, future studies might aim to identify the neuronal proteins that undergo changes in acetylation due to L-lactate induced increased expression of SIRT3. Such data might expand our understanding of the role of L-lactate in brain functions.

It should be noted that L-lactate’s beneficial role is not merely limited to cognitive functions. For example, it was found to be neuroprotective following traumatic brain injury (TBI) (Ichai et al., 2009; Belanger et al., 2011; Bouzat et al., 2014). In TBI, there is energy crisis and increased oxidative stress. Although accumulating evidence suggests that L-lactate acts as an energy molecule to provide neuroprotective effect in TBI (Bouzat et al., 2014; Glenn et al., 2015), it is not clear whether L-lactate can efficiently mitigate oxidative stress in TBI through signaling mechanism. Future studies could aim to delineate whether and how the differentially expressed proteins observed in our study might play role in TBI.

The amount of L-lactate we injected is expected to produce a final average concentration of 5 mM across the injection site. Different studies have used different concentration of L-lactate in different cell lines and subjects including humans and observed varying effects [e.g., reduced intracranial pressure at ≈6 mmol/l blood lactate in TBI patients (Bouzat et al., 2014), neurotoxicity at 20 mmol/l but neuroprotective at 4 mmol/l of L-lactate (Berthet et al., 2009)]. These studies indicate that different concentrations of L-lactate may have different regulatory roles on brain functions under different conditions. Further studies are needed to understand more about the appropriate L-lactate concentration needed to obtain beneficial effect for a given condition.

## Data availability statement

The datasets presented in this study can be found in online repositories. The names of the repository/repositories and accession number(s) are: http://www.proteomexchange.org/, PXD039121 and https://repository.jpostdb.org/, JPST001976.

## Ethics statement

All experimental procedures using animals were conducted according to the guidelines developed by the Committee on Use and Care of Animals, Department of Health, Govt. Hong Kong SAR. The License numbers to conduct experiments are: (22–2) in DH/HT&A/8/2/5 Pt.8 and (22–3) in DH/HT&A/8/2/5 Pt.8. The approval for “Ethical Review of Research Experiments involving Animal Subjects” were taken by Animal Research Ethics Sub-Committee, City University of Hong Kong (References: A-0513 and A-0215).

## Author contributions

MA: conceptualization, methodology, investigation (rat and sample preparation, IHC, WB, confocal imaging, RT-PCR), formal analysis, visualization, and writing—original draft. HM: methodology, investigation (LC–MS/MS), formal analysis, visualization, and writing—original draft. MH: investigation (rat and sample preparation, IHC). AK: methodology, formal analysis, and visualization. XZ: writing—review and editing. LZ: conceptualization, methodology, resources, and supervision. YL: conceptualization, methodology, resources, writing—review and editing, supervision, project administration, and funding acquisition. All authors contributed to the article and approved the submitted version.

## Funding

This research was funded by the General Research Fund (GRF) of the Research Grants Council of Hong Kong (11103721, 11102820, and 11100018), the National Natural Science Foundation of China (NSFC) and RGC Joint Research Scheme (3171101014, N_CityU114/17), the Innovation and Technology Fund Hong Kong (CityU 9445909), the Shenzhen-Hong Kong Institute of Brain Science Innovation Open Project Contract (NYKFKT2019012) and the Shenzhen-Hong Kong Science and Technology Innovation Cooperation Zone Shenzhen Park Project (HZQB-KCZYZ-2021017). This work was also supported by a City University of Hong Kong Neuroscience Research Infrastructure Grant (9610211), and a Centre for Biosystems, Neuroscience, and Nanotechnology Grant (9360148).

## Conflict of interest

The authors declare that the research was conducted in the absence of any commercial or financial relationships that could be construed as a potential conflict of interest.

## Publisher’s note

All claims expressed in this article are solely those of the authors and do not necessarily represent those of their affiliated organizations, or those of the publisher, the editors and the reviewers. Any product that may be evaluated in this article, or claim that may be made by its manufacturer, is not guaranteed or endorsed by the publisher.

## Abbreviations

ACC: Anterior cingulate cortex
ACSF: Artificial cerebrospinal fluid
ANLS: Astrocyte neuron L-lactate shuttle
ETC: Electron transport chain
HPC: Hippocampus
IHC: Immunohistochemistry
I.P.: Intraperitoneal
LCMS: Liquid chromatography mass spectrometry
LDH: Lactate dehydrogenase
MCT: Monocarboxylate transporter
mtDNA: Mitochondrial DNA
nDNA: Nuclear DNA
ROS: Reactive oxygen species
TBI: Traumatic brain injury
WB: Western blot
