# Supplementary material for: Exogenous L-lactate administration in rat hippocampus increases expression of key regulators of mitochondrial biogenesis and antioxidant defense
Source: Front Mol Neurosci. 2023 Mar 16;16:1117146. doi: 10.3389/fnmol.2023.1117146 (PMC10062455; doi:10.3389/fnmol.2023.1117146)
Supplement: Supplementary file 1 [file Data_Sheet_1.docx]

# Exogenous L-lactate administration in rat hippocampus increases expression of key regulators of mitochondrial biogenesis and antioxidant defense

# Supplementary materials


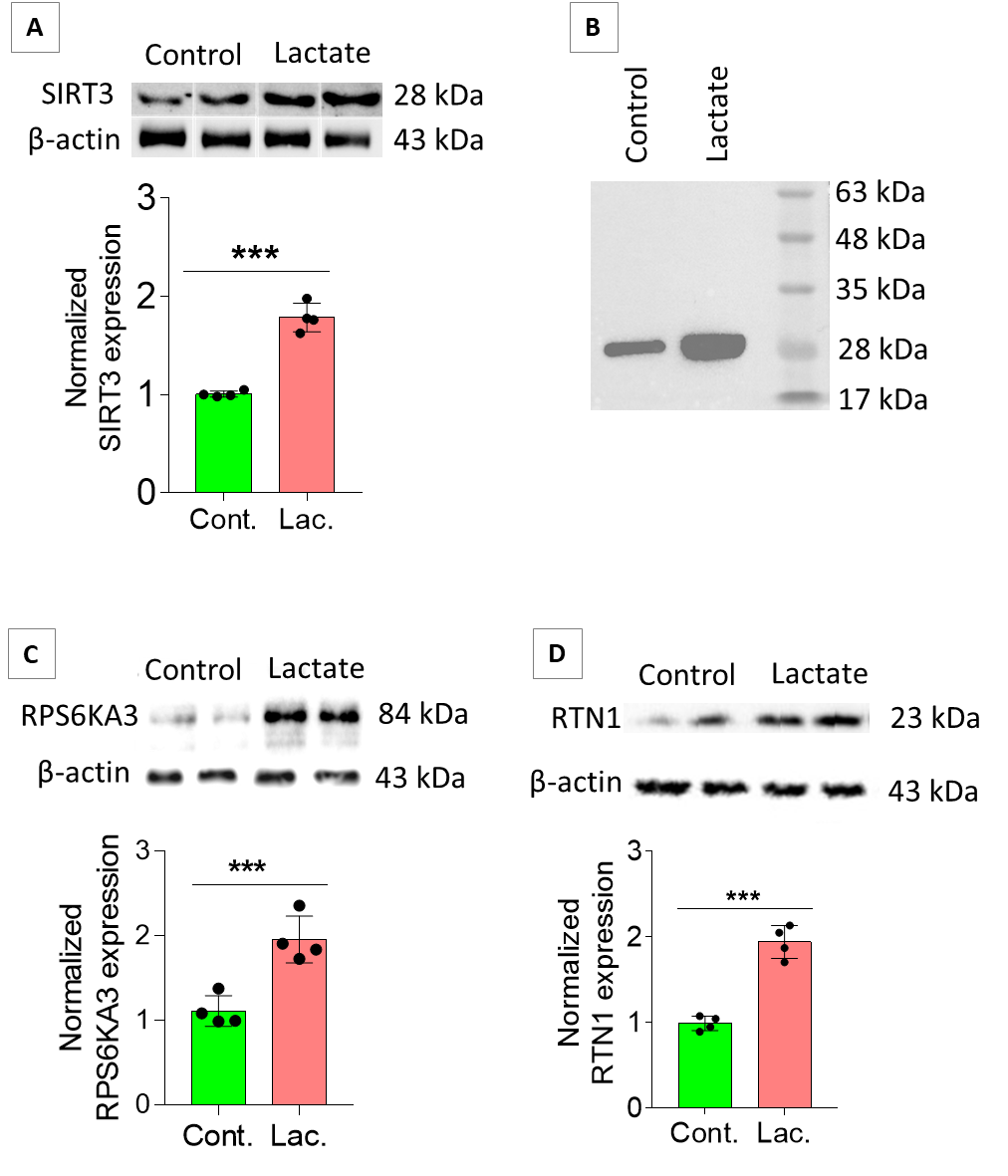


**Supplementary Fig. S1:** Representative WB images of SIRT3 (**A**, **B**), RPS6KA3 (**C**), and RTN1 (**D**) in the HPC extracts from control and lactate groups. Intensity was quantified and normalized with β-actin. Consistent with proteomics data, these proteins were found to be significantly increased in the lactate group compared to control. Data are shown as mean ± SD (n=4 rats per group). *p****<0.001, unpaired Student’s t-test.

**
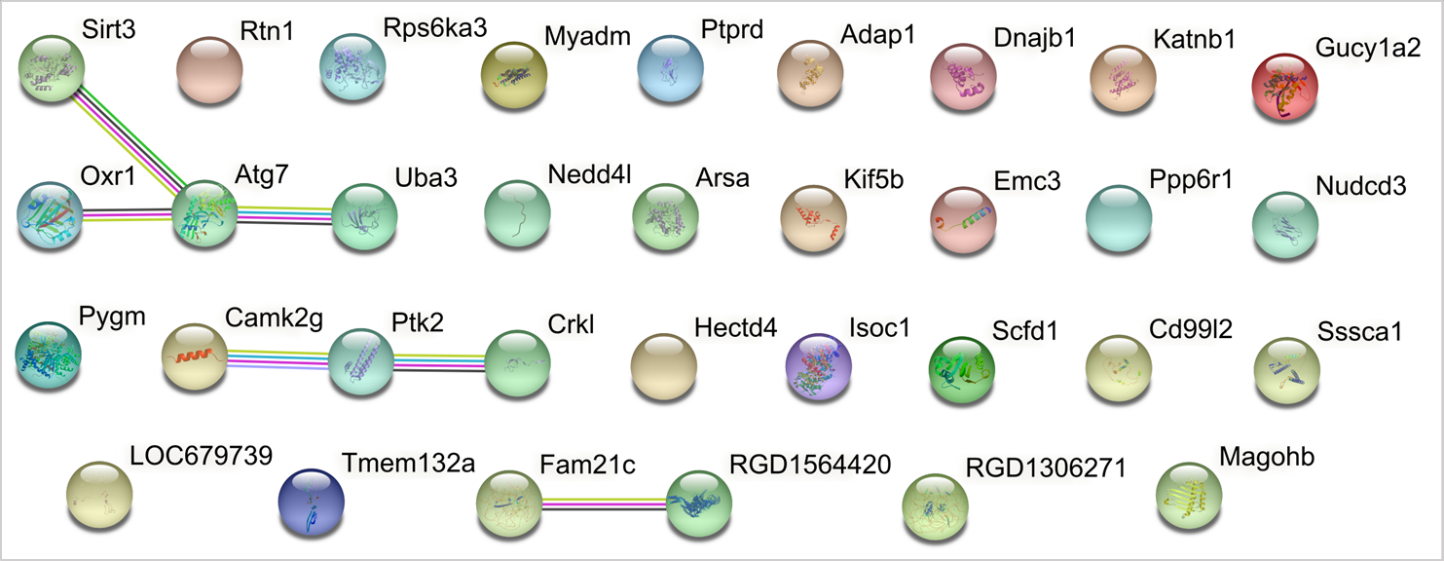
**

**Supplementary Fig. S2**: STRING protein-protein interaction analysis with the upregulated proteins in the lactate group (*p*=0.249).

**
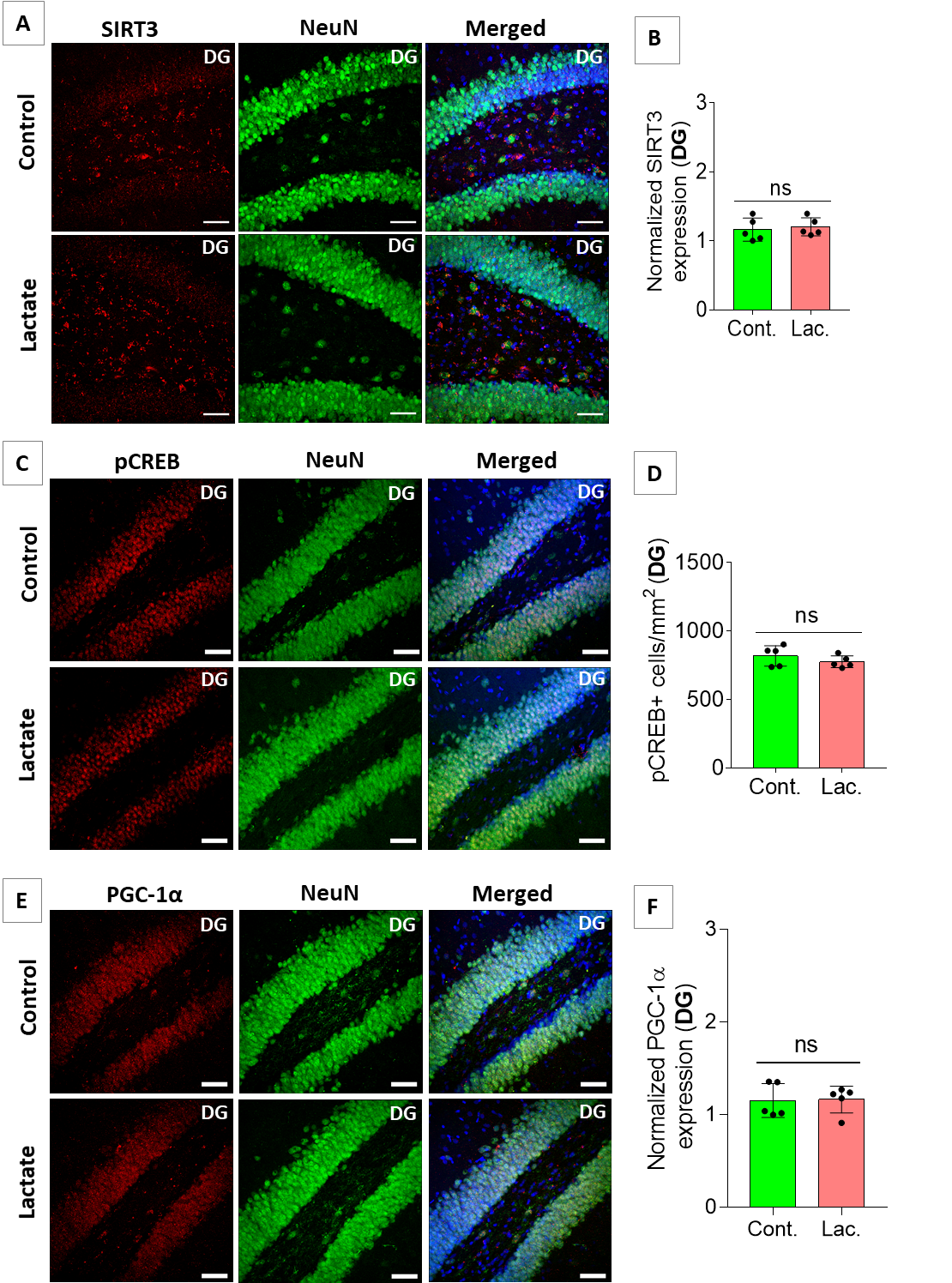
**

**Supplementary Fig. S3**: Expression of SIRT3, pCREB, and PGC-1α in the DG of hippocampus

**A**, **C**, and **E.** Representative confocal micrograph of SIRT3 (red) (**A**) / pCREB (red) (**C**) / PGC-1α (red) (**E**) co-labelled with NeuN (green) in the DG of HPC of control and lactate groups. L-lactate infusion did not increase SIRT3 / pCREB / PGC-1α expression in DG. Scale bars: 50 µm.

**B**, **D, & F**. Fluorescence intensity of SIRT3 / pCREB / PGC-1α stained sections in the DG of HPC of lactate group was assessed and normalized to control group of rats. Data is shown as mean ± SD (n=5 rats per group). ns=not significant, unpaired Student’s t-test.

### Supplementary Table-S1 | Antibodies used in WB and IHC

| **Name of antibodies** | **Source** | **Manufacturer and Catalogue number** | **Dilution** |
| --- | --- | --- | --- |
| Anti-RTN1 | Rabbit | Sigma_SAB2102060 | 1:1000 (WB) |
| Anti-Rsk2/MAPK kinase 1b | Rabbit | Abcam_ab92826 | 1:1000 (WB) |
| Anti-SIRT3 | Rabbit | SAB5700222 | 1:500 (WB), 1:250 (IHC) |
| Anti-ATPB (mitochondrial marker) | Mouse | Abcam_14730 | 1:500 (WB), 1:500 (IHC) |
| Anti-PGC-1α | Rabbit | Abcam_191838 | 1:1000 (WB), 1:500 (IHC) |
| Anti-cytochrome c | Mouse | Abcam_13575 | 1:1000 (WB), 1:500 (IHC) |
| Anti-β-actin | Mouse | Immunoway_YM3028 | 1:5000 (WB) |
| Anti-pCREB | Rabbit | EMD Millipore corp. USA, 06-519 | 1:500 (IHC), 1:1000 (WB) |
| Anti-CREB | Rabbit | Sigma_04-767 | 1:1000 (WB) |
| Anti-GFAP | Chicken | EMD Millipore corp. USA, AB5541 | 1:500 (IHC) |
| Anti-NeuN | Rabbit | EMD Millipore corp. USA, AB978 | 1:500 (IHC) |
| Anti-GAPDH | Mouse | Abcam (8245) | 1:1000 (WB) |
| Alexa Flour 488, 594 goat anti mouse IgG (H+L) | Goat | Thermo fisher scientific_A11001, Thermo fisher scientific_A11032 | 1:300 (IHC) |
| Alexa Flour 488, 594 goat anti rabbit IgG (H+L) | Goat | Thermo fisher scientific­_A11034, Thermo fisher scientific_A11037 | 1:300 (IHC) |
| Goat anti-mouse and rabbit secondary antibody, HRP | Mouse, Rabbit | Invitrogen_31460 (rabbit), 62-6520 (mouse) | 1:5000 (WB) |

### Supplementary Table-S2 | Reactome pathway enrichment analysis with the upregulated proteins in the L-lactate treated rats

| **Reactome pathways** | **Fold Enrichment** | **Raw *p* value** |
| --- | --- | --- |
| Regulation of FOXO transcriptional activity by acetylation | > 100 | 1.03E-02 |
| 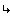FOXO-mediated transcription | 32.12 | 3.21E-02 |
| The activation of arylsulfatases | 74.95 | 1.47E-02 |
| 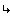Post-translational protein modification | 2.77 | 3.23E-02 |
| MET receptor recycling | 67.45 | 1.62E-02 |
| 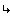Signaling by MET | 17.52 | 6.09E-03 |
| Erythropoietin activates RAS | 67.45 | 1.62E-02 |
| 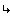Signaling by Erythropoietin | 39.68 | 2.63E-02 |
| RHO GTPases activate KTN1 | 61.32 | 1.76E-02 |
| 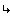Signaling by Rho GTPases | 4.34 | 1.32E-02 |
| 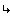Signaling by Rho GTPases, Miro GTPases and RHOBTB3 | 4.24 | 1.43E-02 |
| MET activates RAP1 and RAC1 | 61.32 | 1.76E-02 |
| 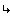MET promotes cell motility | 34.59 | 1.68E-03 |
| DCC mediated attractive signaling | 61.32 | 1.76E-02 |
| 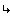Netrin-1 signaling | 26.98 | 3.78E-02 |
| Frs2-mediated activation | 56.21 | 1.91E-02 |
| 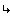Prolonged ERK activation events | 48.18 | 2.20E-02 |
| 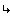Signalling to ERKs | 24.98 | 4.07E-02 |
| Glycogen breakdown (glycogenolysis) | 56.21 | 1.91E-02 |
| 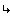Glycogen metabolism | 39.68 | 2.63E-02 |
| Transcriptional activation of mitochondrial biogenesis | 51.89 | 2.05E-02 |
| 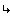Mitochondrial biogenesis | 21.76 | 4.63E-02 |
| Attenuation phase | 51.89 | 2.05E-02 |
| 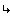HSF1-dependent transactivation | 58.65 | 6.23E-04 |
| 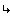Cellular response to heat stress | 15.87 | 7.34E-03 |
| GRB2:SOS provides linkage to MAPK signaling for Integrins | 48.18 | 2.20E-02 |
| 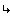Integrin signaling | 25.94 | 3.92E-02 |
| p130Cas linkage to MAPK signaling for integrins | 48.18 | 2.20E-02 |
| Interferon gamma signaling | 39.68 | 2.63E-02 |
| 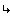Cytokine Signaling in Immune system | 5.37 | 1.83E-02 |
| Receptor-type tyrosine-protein phosphatases | 35.50 | 2.92E-02 |
| Unblocking of NMDA receptors, glutamate binding and activation | 33.73 | 3.06E-02 |
| 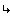Activation of NMDA receptors and postsynaptic events | 21.76 | 4.63E-02 |
| Synaptic adhesion-like molecules | 32.12 | 3.21E-02 |
| Regulation of signaling by CBL | 32.12 | 3.21E-02 |
| Downregulation of SMAD2/3:SMAD4 transcriptional activity | 29.33 | 3.49E-02 |
| MET activates PTK2 signaling | 24.09 | 4.21E-02 |
| Downstream signal transduction | 23.26 | 4.35E-02 |
| NCAM signaling for neurite out-growth | 21.76 | 4.63E-02 |
| Phase 0 - rapid depolarisation | 21.08 | 4.77E-02 |
| COPII-mediated vesicle transport | 20.14 | 4.68E-03 |
| 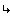ER to Golgi Anterograde Transport | 9.37 | 1.97E-02 |
| 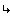Transport to the Golgi and subsequent modification | 7.80 | 2.76E-02 |
| 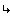Membrane Trafficking | 3.69 | 4.73E-02 |
| RHOA GTPase cycle | 9.85 | 1.79E-02 |
| Ion channel transport | 7.98 | 2.64E-02 |
| Antigen processing: Ubiquitination & Proteasome degradation | 6.95 | 9.23E-03 |
| 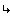Class I MHC mediated antigen processing & presentation | 5.90 | 1.43E-02 |
| 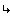Adaptive Immune System | 3.60 | 2.42E-02 |
| MAPK family signaling cascades | 6.81 | 9.75E-03 |
| Innate Immune System | 2.90 | 4.75E-02 |
